# Reusability and regeneration of solid catalysts used in ultrasound assisted biodiesel production

**DOI:** 10.3906/kim-2008-33

**Published:** 2021-04-28

**Authors:** Mahmut BAYRAMOĞLU, İbrahim KORKUT, Başak TEMUR ERGAN

**Affiliations:** 1 Chemical Engineering Department, Gebze Technical University, Gebze Turkey; 2 Chemical Engineering Department, Sivas University of Science and Technology, Sivas Turkey

**Keywords:** Biodiesel, ultrasound assisted transesterification, calcium oxide, calcined dolomite, heterogeneous catalyst regeneration

## Abstract

Reusability of two heterogeneous catalysts in ultrasound (US) assisted biodiesel production was investigated in comparison to each other. An ultrasound (US) generator (200 W, 20 kHz) equipped with a horn type probe (19 mm) was used. Regeneration experiments were planned according to second order central composite design (CCD) method. After the eighth use of the catalysts, biodiesel yield decreased from 99.1% to 90.4% for calcined calcite (CaO) and from 98.8% to 89.8% for calcined dolomite (CaO.MgO). Furthermore, regeneration of spent catalysts by calcination was investigated; optimum temperature and time were found as 750 °C and 90 min, lower than fresh catalyst preparation conditions. The regenerated catalysts were reused in a second process cycle; biodiesel yield was calculated as 97.2% for CaO and 96.5% for CaO.MgO. Finally, the process showed that calcination is an energetically favorable regeneration process of spent catalysts.

## 1. Introduction

Transesterification reaction transforms oil or fats with methanol (CH3OH) into biodiesel in presence of a catalyst, with glycerol as a by-product [1,2]. Usually, homogeneous basic catalysts such as potassium hydroxide (KOH) or homogeneous acidic catalysts such as sulfuric acid (H_2_SO_4_) are used [3]. However, the homogeneously catalyzed transesterification process has some drawbacks such as the formation of soap and water, which consume more catalyst and reduce the biodiesel yield and the biodiesel quality. Furthermore, additional water is spent for purification of biodiesel and glycerol and more wastewater is formed, disposal of which increases the process cost [4]. The heterogeneously catalyzed transesterification process exhibits various advantages; the catalyst can be easily removed by filtration from the reaction medium, the process includes fewer number of unit operations, more pure products (biodiesel and glycerol) are obtained, wastewater formation during product purification steps is greatly reduced, heterogeneous catalyst is reused until a catalyst regeneration step, which reduces biodiesel production cost [5]. Meanwhile, the major drawback of heterogeneous catalysis is the slow reaction rate compared to homogeneous counterpart. Fortunately, the process can be accelerated by applying ultrasound in the reaction medium [1,6]. 

In virtue of these advantages, studies on biodiesel production in the presence of heterogeneous catalysts have been increasing over the last 10 years [6]. The commercialization of heterogeneously catalyzed biodiesel production requires profitability, raw material availability, and cost-effective production [7]. In this respect, the reusability of catalyst is a very important issue that should be examined, which has not been investigated in detail except a few studies without any mention about the catalyst regeneration; Piker et al. reused the CaO catalyst ten times with fresh oil and four times with waste cooking oil at the same reaction conditions; biodiesel yield decreased from 95% to 75% in the case of fresh oil and decreased from 93% to 62% in case of using waste cooking oil [8]. Viola et al. investigated the catalytic activity of CaO in the reuse experiments and found that the loss of efficiency was due to the attached glycerin on the catalyst surface [9]. Kurayama et al. used Ca-loaded microcapsules as catalyst for the transesterification of rapeseed oil, and they found that the biodiesel yield declined from 95.5% to 80.9% at the end of fourth reuse [10]. Castro et al. prepared CaO/MgAl oxide catalysts, and they found that the catalyst could be reused for at least 5 reuse cycles without effective decline of the catalyst activity [11]. Wen et al. prepared KF/CaO nano catalyst by impregnation method for transesterification of Chinese tallow seed oil and reused the catalyst 16 times with a slight loss of biodiesel yield [12]. Hu et al. prepared a nanomagnetic catalyst, KF/CaO–Fe_3_O_4_, using an impregnation method for biodiesel production and reused the catalyst 16 times [13]. Yu et al. synthesized different compositions of CaO–CeO_2_ mixed metal oxides for using solid base catalysts and found that the biodiesel yield decreased slowly until the fifth reuse when a significant loss in the catalytic activity was observed [14]. 

On the other hand, catalyst regeneration methods can be classified into two groups. In the first group, after catalyst filtration, regeneration is accomplished by washing and drying the catalyst at a temperature usually lower than 200 °C. In the second one, calcination alone or combined with other methods is applied [6]. Madhu et al. found that reused catalysts must be calcinated in order to activate the catalyst active sites [15]. Boey et al. reused the waste cockle shell derived CaO catalyst at least for three times, with a purity above 96.5%. Before it was reused, the spent catalyst was washed with methane and n-hexane to remove the adsorbed materials and calcined at 900 °C for 2 h. The washed uncalcined spent catalyst consisted of Ca(OH)_2_ and traces of Ca(C_3_H_7_O_3_)_2_ (calcium diglyceride, CaDG). After calcination at 900 °C for 2 h, the structure of the calcined dolomite catalyst was changed to CaO [16]. 

The literature survey shows that calcination is a more appropriate method to regenerate the activity of CaO and Ca based catalysts; it is a simple process with no waste solvent or solid waste product formation, also the heat of combustion of some organic compounds formed during the esterification such as CaDG or adsorbed impurities on the spent catalyst may diminish the heat duty of the calcination process. 

By considering these points, calcination was selected in this study for catalyst regeneration, which it is expected to be more energetically favorable process compared to fresh catalyst preparation.

## 2. Experimental section

### 2.1. Chemicals

Commercial canola oil with the following relevant properties was used without purification: free acid content: 0.5%; density: 0.93 g/cm3. Kinematic viscosity: 77 mm2/s; unsaponifiables: 0.7%; triglycerides: 97.2% of total lipids. Dolomite (CaCO_3_.MgCO_3_) and calcite (CaCO_3_) were obtained from chemical supplier located in Kocaeli, Turkey. They were calcined at 840 °C for 3 h [17].

### 2.2. Catalyst reuse experiments 

The schematic representation and details of the experimental setup were given in a previous study [1]; an ultrasonic generator (Bandelin 2200 sonopuls, 200 W, 20 kHz, Sigma–Aldrich, city, country?) equipped with a horn type probe was used to deliver pulsed ultrasound (US) with controllable power in a 300 mL three-necked cylindrical glass reactor equipped with a reflux condenser and a magnetic stirrer.

The experiments were carried out at the optimum conditions determined in this study. At the end of the run, the reaction slurry was poured into specially designed bottom screwed centrifuge tubes, which allowed the catalyst recovery almost completely? Until the next run, the catalyst was kept in refrigerator in CH_3_OH to avoid contact with atmospheric CO_2_ and H_2_O. The next run required CH_3_OH for the desired feed CH_3_OH/oil molar ratio, and oil was added to the sonoreactor with some fresh catalyst to compensate for the catalyst loss (approximately 5%).

### 2.3. Catalyst regeneration experiments

The purpose of these experiments was to find calcination conditions energetically (and economically) favorable than the fresh catalyst production by calcination from raw materials. Catalysts** (**obtained after the eighth use) were divided into samples of 1-g after washing with methanol. Catalyst regeneration experiments were carried out using 1-g catalyst samples. The statistical second order central composite (CCD) design was used in planning of regeneration experiments using Design-Expert software (demo version, Stat-Ease Inc, MN, USA?). In addition, temperature and time were selected as process factors in the experimental plan; as seen in Table 1, positive levels of factors were determined from fresh catalyst preparation conditions, and the negative levels were determined from similar catalyst regeneration studies cited in the literature [18,19].

**Table 1:** Factor levels for central composite experimental design.

Factor	–1	+1	–α	+α
Temperature (°C)	550	750	450	850
Time (min)	90	150	60	180

The second order central composite (CCD) experimental plan is given in Table 2. Eleven regeneration experiments were made for this purpose. The response of the experimental plan was the mass loss (%) of the spent catalyst at the end of the calcination, high mass loss indicating the success of the calcination. The standard deviation of mass loss calculated from the center point experiments results was found as 0.27%.

**Table 2:** Experimental plan and mass loss of the spent catalysts.

Temperature (°C)	Time (min)	Mass loss (%)
450	120	54.56
550	90	56.99
550	150	57.24
650	60	59.48
650	120	64.64
650	120	64.30
650	120	64.84
650	180	66.67
750	90	74.00
750	150	74.60
850	120	74.60

## 3. Results and discussion

### 3.1. Catalyst reuse experiments 

**Figure 1:**
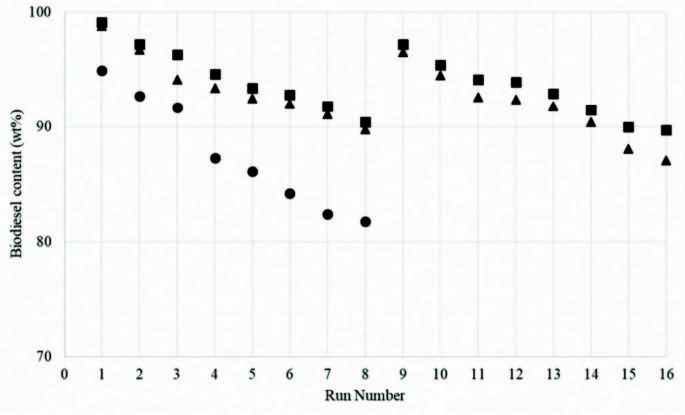
Reusability of catalysts in the transesterification of canola oil. (▀ CaO catalyst ultrasound assisted transesterification experiments, ▲calcined dolomite ultrasound assisted transesterification and ● CaO silent transesterification experiments).

**Figure 2:**
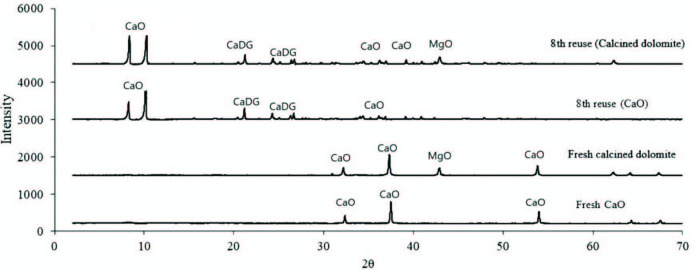
The XRD patterns of fresh and used catalysts.

The serial experiments were carried out until the yield decreased to approximately 90%. Experimental results are depicted in Figure 1. Different heterogeneous catalyst deactivation mechanisms are proposed in literature; Oueda, et al. pointed out especially to the leaching of active sites on the catalyst surface, the surface poisoning and/or pore filling, and the structural collapse of catalysts [6]. To detect phase transformation during the process, X-ray diffraction (XRD) patterns of fresh and spent catalysts with crystal phases are shown in Figure 2; as also noted by Granados et al. the oxide form disappears in both catalysts and new peaks arise which show that CaO is transformed to CaDG (the characteristic XRD peaks of CaDG at 2θ are 8.1°, 10.1°, 21.1°, 24.2°, 26.6°, 34.3° and 36s.4°) according to the reaction in Equation (1) [20].

(1)CaO(s)+2C3H8O3(l)→Ca(C3H7O3)2(s)+H2O(l)

CaDG is less active transesterification catalyst than CaO catalyst. Furthermore, CaDG is more soluble in the glycerol phase than CaO. Also, water reacts with CaDG according to reaction in Equation (2).

(2)Ca(C3H7O32(s)+2H2O(l)

Furthermore, Ca^2+^ reacts with the free fatty acid (FFA) in the oil and forms calcium soaps Ca(FFA)_2_ according to the reaction in Equation (3), which results in the reduction of the reaction yield and biodiesel purity.

(3)2FFA+CAO(s)↔Ca(FFA)2+H2O

### 3.2. Catalyst regeneration experiments

The XRD data was used to check the success of calcination procedures and synthesis of CaDG. As seen in Figure 2, sharp and highly intense XRD peaks define the well crystallized structure of the regenerated spent catalysts. Because, the crystallinity or crystallite size of the catalyst is an important feature determining the catalytic activity. Therefore, the crystallite size for all phases was obtained from the XRD patterns using the Scherrer equation (Equation 4) [21–23]. 

(4)L=αλβ cosθ

where, L is the mean average crystallite size (Å), α is a constant equal to 0.94, β is the full width at the half maximum in radians (obtained for the high intensity peak), and λ (Å) is the wavelength of the X-rays (1.54059 Å). For eighth reuse calcined dolomite (CaO.MgO), eighth reuse calcined calcite (CaO), fresh calcined dolomite (CaO.MgO) and fresh calcined calcite (CaO), the average crystallite size values were 64.4 Å, 99.0 Å, 70.6 Å, and 105.8 Å, respectively. In this study, the maximum average crystallite size was observed at fresh calcined calcite (CaO). Crystallite size values in optimum calcination conditions of the fresh and used catalyst are shown in Table 3.

**Table 3:** The crystallite size of the fresh and used catalysts.

Catalysis	Temperature (°C)	Time (min)	Crystallite size (Å)
8th CaO.MgO	750	90	64.4
8th CaO	750	90	99.0
Fresh CaO.MgO	840	180	70.6
Fresh CaO	840	180	105.8

When CaDG is completely oxidized to CaO, a mass loss of 74.7% occurs according to the reaction in Equation (5).

(5)Ca(C3H7O32(s)+7O2→CaO+7H2O+6CO2

Thus, the highest mass loss in Table 2 (74.6%) refers to optimum regeneration conditions; 750 °C and 90 min. which are sufficient to convert CaDG completely to CaO. 

In the second cycle of the biodiesel production with regenerated catalyst, yield was realized as 97.2% for CaO. For dolomite catalyst the same calcination conditions was used for regeneration step and 96.5% yield was obtained. 

**Figure 3:**
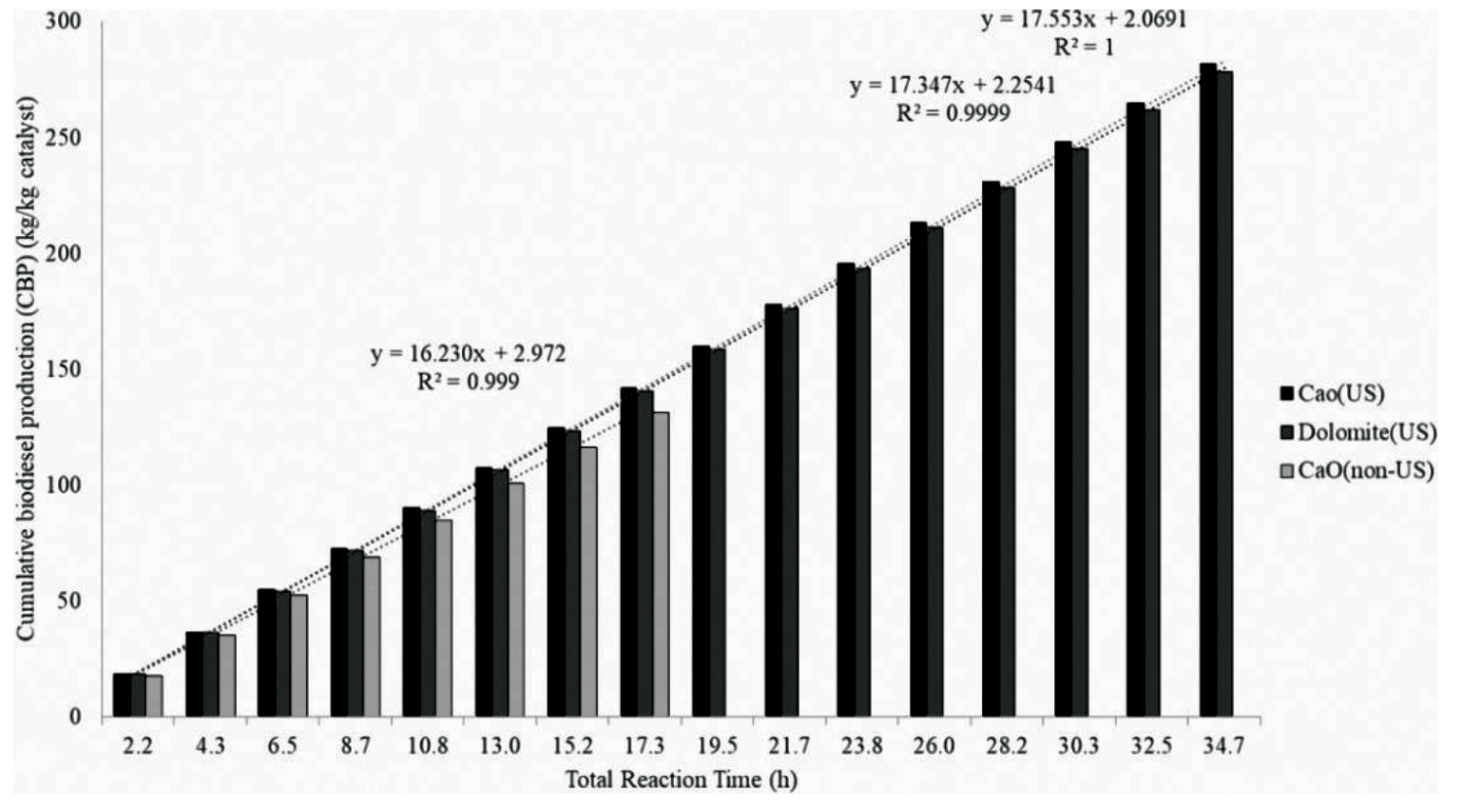
Cumulative biodiesel production as a function of total rreaction time.

Finally, for an overall assessment of the process, biodiesel production per kg of catalyst (BP) was calculated by Equation (6), where moil is amount of canola oil (kg), α is the stoichiometric correction factor, x is the biodiesel content of product and mcatalyst is the amount of used catalyst (kg). The cumulative biodiesel production per kg of catalyst value (CBP) is obtained by accumulating the BP values. The CBP results are given in Figure 3. As seen, the CBP values of both catalysts are very close to each other. 

(6)BP=moilα xmcatalyst

On the other hand, cumulative biodiesel production rate (BPR) is obtained by dividing CBP values by reaction time. BPR values (kg biodiesel/kg catalyst h) were calculated as 17.55, 17.35, and 16.23 for CaO (US), calcined dolomite (US), and CaO (silent) respectively. The cumulative productivity rate of US assisted biodiesel production is 8% higher than the silent biodiesel production, but for a more detailed energy analysis of the biodiesel production process, the comparison may be more accurate by taking into account ultrasound energy used during the reaction.

## 4. Conclusion

The reusability performances of calcined calcite (CaO) and calcined dolomite (CaO.MgO) in the ultrasound assisted transesterification reactions were tested in successive transesterification runs. The yields decreased from 99.1% and 98.8% for CaO and calcined dolomite to 90.4% and to 89.8% after eighth reuse, respectively. These results are consistent with the average crystallite size results and also with XRD patterns.

The regenerated catalysts were reused in a second process cycle with negligible activity losses compared to fresh catalysts. Furthermore, an overall assessment of the process by means of various criteria shows that the performance of ultrasound assisted biodiesel production using CaO catalyst is 8% higher than the silent biodiesel production.

While the use of ultrasound in biodiesel production decreases the process cost and process time, the eighth reuse of calcined calcite and calcined dolomite catalysts reduces the energy requirement in catalyst preparation.

 In conclusion, the experimental results confirmed that spent catalyst calcination at 750 °C for 90 min. is more energetically favorable when compared to fresh catalyst preparation at 840 °C for 3 h. Finally, reusability of heterogeneous catalyst is an important issue affecting technical, environmental and economic aspects of the biodiesel production process.
